# 

**Published:** 1903-09-15

**Authors:** 


### PRELIMINARY ANNOUNCEMENT OF THE FOURTH INTERNATIONAL DENTAL CONGRESS.

To be held August 29 to September 3, 1904, in connection with the Universal Exposition, St. Louis, 1904.

The Department of Congresses of the Universal Exposition, St. Louis, has nominated the Committee of Organization of the Fourth International Dental Congress, and has instructed the Committee thus appointed to proceed with the work of organization of said Congress.

Pursuant to the instructions of the Director of Congresses of the Universal Exposition, 1904, the Committee of Organization presents the subjoined outline of the plan of orgazation of the Dental Congress.

The Congress will be divided into two departments: Department A—SCIENCE (divided into four sections). Department B—APPLIED SCIENCE (divided into six sections).

### DEPARTMENT A—SCIENCE.

I. Anatomy, Physiology, Histology and Microscopy; Chairman, M. H. Cryer. II. Etiology, Pathology and Bacteriology; Chairman, R. H. Hofheinz. III. Chemistry and Metallurgy; Chairman, J. D. Hodgen. IV. Hygiene, Prophylaxis, Therapeutics, Materia Medica and Electro-therapeutics; Chairman, A. H. Peck.

### DEPARTMENT B—APPLIED SCIENCE.

V. Oral Surgery; Chairman, G. V. I. Brown. VI. Orthodontia; Chairman, E. H. Angle. VII. Operative Dentistry Chairman, C. N. Johnson: VIII. Prosthesis; Chairman, C. R. Turner. IX. Education, Nomenclature, Literature and History; Chairman, T. W. Brophy. X. Legislation; Chairman, Wm. Carr.

### COMMITTEES.

The following Committees were appointed: Finance; Chairman, C. S. Butler. Program; Chairman, A. H. Peck. Exhibits; Chairman, D. M. Gallie. Transportation; (To be appointed.) Reception; Chairman, B. Holly Smith. Registration; Chairman, B. L. Thorpe. Printing and Publication; Chairman, W. E. Boardman. Conference with State and Local Dental Societies; Chairman, J. A. Libbey. Dental Legislation; Chairman, Wm. Carr. Auditing; (Committee of Organization.) Invitation; Chairman, L. G. Noel. Membership; Chairman, J. D. Patterson. Educational

Methods; Chairman, T. W. Brophy. Oral Surgery; Chairman, G. V. I. Brown. Prosthetic Dentistry; Chairman, C. R. Turner. Local Committee of Arrangements; (To be appointed.) Essays; (To be appointed.) History of Dentistry; Chairman, Wm. H. Trueman. Nomenclature; Chairman, S. W. Foster. Promotion of Appointment of Dental Surgeons in the Armies and Navies of the World; Chairman, Wm. Donnally. Care of the Teeth of the Poor; Chairman, Thomas Fillebrown. Etiology, Pathology and Bacteriology; Chairman, R. H. Hofheinz. Prize Essays; Chairman, James Truman. Oral Hygiene, Prophylaxis, Materia Medica, Therapeutics and Electro-therapeutics; Chairman, A. H. Peck. Operative Dentistry; Chairman, C. N. Johnson. Resolutions; Chairman, J. Y. Crawford. Clinics; Chairman, C. E. Bentley. Nominations; (To be appointed.) Local Reception Committee; (To be appointed.) *Ad interim;* Chairman, G. V. I. Brown.

The officers of the Congress, president, vice-presidents, secretary and treasurer, will be elected by the Congress at large at the time of the meeting, and will be nominated for the several positions by the nominating committee.

The Congress will be representative of the existing status of dentistry throughout the world. It is intended further that it shall set forth the history and material progress of dentistry from its crude beginnings, through its several developmental stages, up to the present condition.

The International Dental Congress is but one of the large number of congresses to be held during the period of the Louisiana Purchase Exposition, and these in their entirety are intended to exhibit the intellectual progress of the world, as the Exposition will set forth the material progress which has taken place since the Columbian Exposition in 1893.

It is important that each member of the dental profession in America regard this effort to hold an International Dental Congress as a matter in which he has an individual interest and one which he is under obligation to personally help toward a successful issue. The dental profession of America has not only its own professional record to maintain with a just pride, but, as it is called upon to act the part of host in a gathering of our colleagues from all parts of the world, it has to sustain the reputation of American hospitality as well.

The Committee of Organization appeals earnestly to each member of the profession to do his part in making the Congress a success. Later bulletins will be issued setting forth the personnel of the organization and other particulars, when the details have been more fully arranged.

H. J. BURKHART, *Chairman of Committee of Organization.*

E. C. KIRK, *Secretary.*

*Approved:* HOWARD J. ROGERS, *Director of Congresses.*
DAVID R. FRANCIS, *President of the Exposition.*

